# Supplementary material for: Functional Delineation of a Protein–Membrane Interaction Hotspot Site on the HIV-1 Neutralizing Antibody 10E8
Source: Int J Mol Sci. 2022 Sep 15;23(18):10767. doi: 10.3390/ijms231810767 (PMC9504841; doi:10.3390/ijms231810767)
Supplement: Supplementary file 1 [file ijms-23-10767-s001.zip › ijms-1899680-supplementary.pdf]

SUPPLEMENTARY MATERIALS

FUNCTIONAL DELINEATION OF A PROTEIN-MEMBRANE INTERACTION HOTSPOT SITE ON THE HIV-1 NEUTRALIZING ANTIBODY 10E8

Sara Insausti[1,2], Miguel Garcia-Porras[1], Johana Torralba[1,2], Izaskun Morillo[1], Ander Ramos-Caballero[1], Igor de la Arada[1], Beatriz Apellaniz[3], Jose M.M. Caaveiro[4], Pablo Carravilla[5], Christian Eggeling[5,6,7], Edurne Rujas[8,9,10*], and Jose L. Nieva[1,2*]

[1]Instituto Biofisika (CSIC-UPV/EHU), University of the Basque Country (UPV/EHU), PO Box 644, 48080 Bilbao, Spain.

[2]Department of Biochemistry and Molecular Biology, University of the Basque Country (UPV/EHU), PO Box 644, 48080 Bilbao, Spain.

[3]Department of Physiology, Faculty of Pharmacy, University of the Basque Country (UPV/EHU), Paseo de la Universidad, 7, 01006 Vitoria-Gasteiz, Spain.

[4]Laboratory of Global Healthcare, School of Pharmaceutical Sciences, Kyushu University, Fukuoka 819-0395, Japan

[5]Leibniz Institute of Photonic Technology e.V., Jena, Germany

[6]Faculty of Physics and Astronomy, Institute of Applied Optics and Biophysics, Friedrich Schiller University Jena, Jena, Germany

[7]Medical Research Council Human Immunology Unit, Weatherall Institute of Molecular Medicine, University of Oxford, Oxford, United Kingdom

[8]Ikerbasque, Basque Foundation for Science, 48013, Bilbao, Spain

[9]Pharmacokinetic, Nanotechnology and Gene Therapy Group, Faculty of Pharmacy, University of the Basque Country UPV/EHU, 01006 Vitoria-Gasteiz, Spain

[10]Bioaraba, Microbiology, Infectious Disease, Antimicrobial Agents, and Gene Therapy, 01006 Vitoria-Gasteiz, Spain

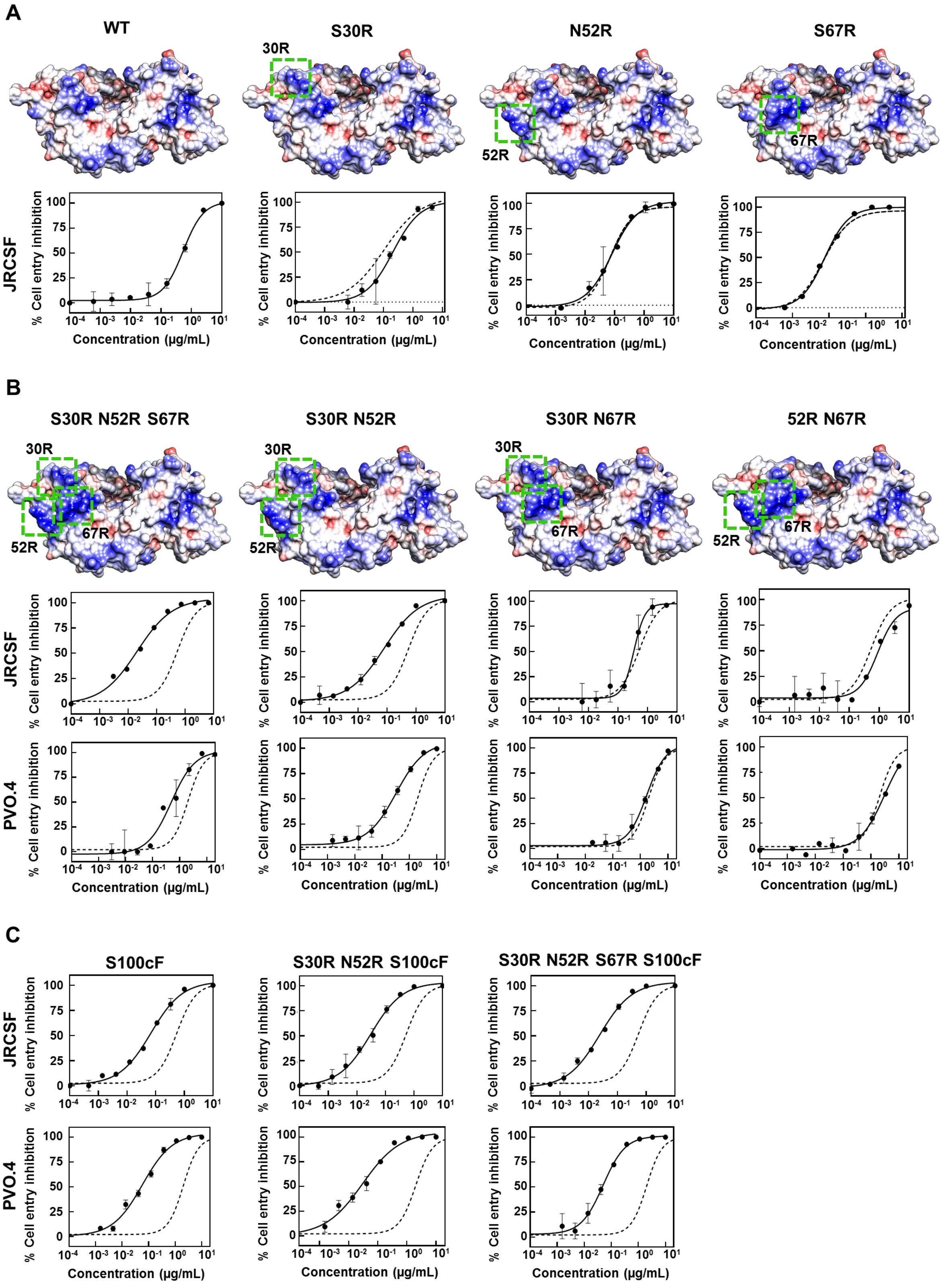
A
WT
S30R
N52R
S67R
30R
52R
67R
JRCSF
% Cell entry inhibition
Concentration (µg/mL)
B
S30R N52R S67R
S30R N52R
S30R N67R
52R N67R
JRCSF
PVO.4
C
S100cF
S30R N52R S100cF
S30R N52R S67R S100cF
JRCSF
PVO.4

Supplementary Figure S1 (previous page): Cell-entry inhibition by 10E8 Fab mutants. Surface density charge representation and neutralization curves of individual (A) and combined (B) Arg mutants. The regions where the mutations are located are highlighted with a green box. C) Neutralization curves of S100cF substitution-containing mutants. 10E8-WT Fab is shown as a dotted line in each plot for comparison.

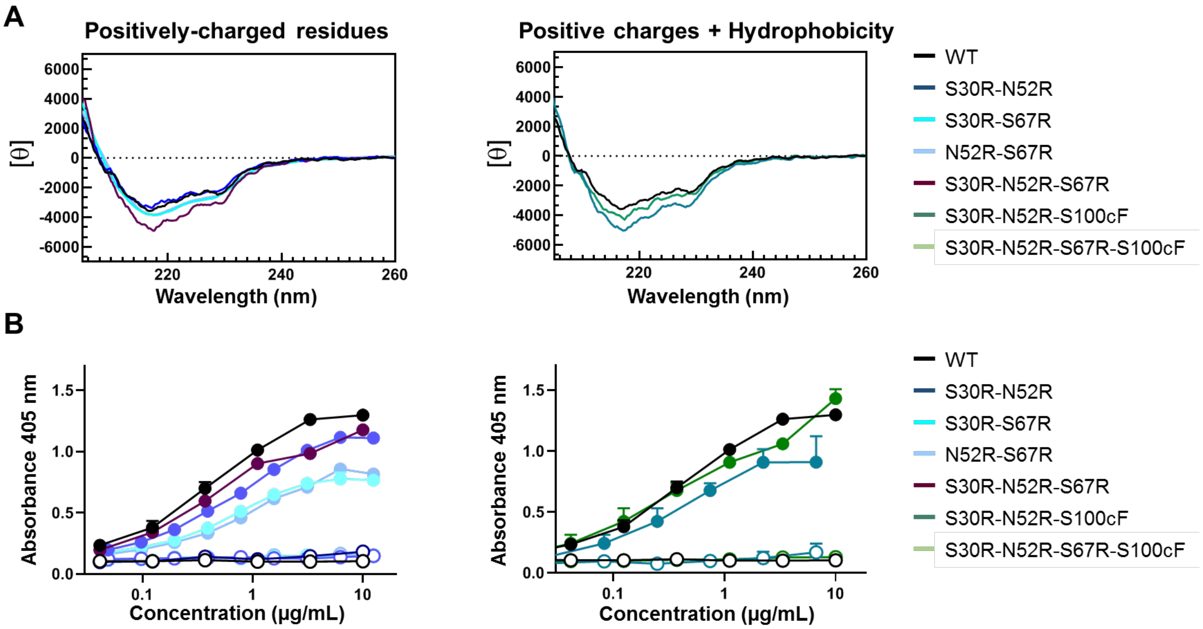

Supplementary Figure S2: Characterization of 10E8 mutant Fabs. (A) Secondary structure by CD and (B) ELISAs to determine functional binding to $MPER_{671-693}$ epitope peptide (filled symbols) and $_{mut}MPER_{671-693}$ peptide (empty symbols), the latter included as a control for binding specificity.

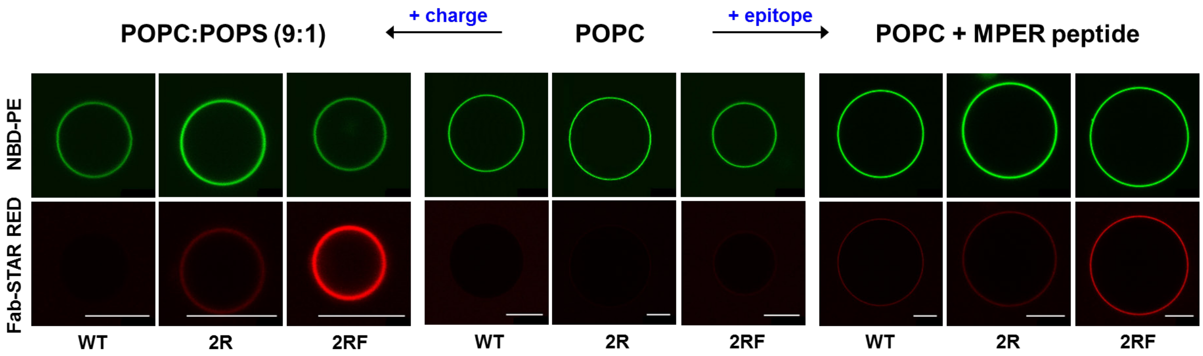

Supplementary Figure S3: Representative images of POPC vesicle binding experiments in the absence (middle) and presence (right) of epitope, and in vesicles including the anionic phospholipid POPS without epitope (left). Scale bars, 5 µm

Supplementary Table S1: Kinetic parameters of binding of Fab 10E8 variants to MPER-$TMD_{671-709}$ peptide reconstituted in SLBs

**Fab**	$\boldsymbol{K_D}$ **(M)**	$\boldsymbol{k_a}$ **(M^{-1} s^{-1}))**	$\boldsymbol{k_{off}}$ **(s^{-1})**
WT	$1.0 \cdot 10^{-9} \pm 0.3 \cdot 10^{-9}$	$1.9 \cdot 10^{6} \pm 0.3 \cdot 10^{6}$	$1.9 \cdot 10^{-3} \pm 0.6 \cdot 10^{-3}$
2R	$1.1 \cdot 10^{-9} \pm 0.4 \cdot 10^{-9}$	$1.5 \cdot 10^{6} \pm 0.5 \cdot 10^{6}$	$1.6 \cdot 10^{-3} \pm 0.1 \cdot 10^{-3}$
2RF	$6.3 \cdot 10^{-10} \pm 5 \cdot 10^{-10}$	$7.3 \cdot 10^{5} \pm 6 \cdot 10^{5}$	$5.1 \cdot 10^{-4} \pm 6 \cdot 10^{-4}$
